# 

**Published:** 1904-11-05

**Authors:** 


### NOTICE TO CORRESPONDENTS.

All MSS., Letters, Books for Review, and other matters intended for the Editor should be addressed The Editor, "The Hospital," The Hospital Buildings, 28 & 29 Southampton Street, Strand, W.C.

The Editor cannot undertake to return rejected MS., even when accompanied by stamped directed envelope.

### Notice to Advertisers and Subscribers.

All Advertisements, Orders for Copies of The Hospital, and Business Communications should be addressed to **THE MANAGER (Not to the Editor)**, The Hospital, 28 & 29 Southampton Street, Strand, London, W.C.

The Cost of Subscription, post free, is as follows:—Per week, 3½d.; per quarter, 3s. 3d.; per half-year, 6s. 6d.; per annum, 13s. Foreign and Colonial:—Per annum, 19s. 6d., payable, in all cases, in advance.

**NOTICE.—Vol. XXXVI. of "The Hospital" (April to September 1904), in handsome cloth binding, is now on sale at the office of this Journal, price 10s. 6d. Cases for binding the Numbers contained in this Volume 2s. Index to Vol. XXXVI., 3½d., post free.**

**The Monthly part for November is now ready. Price 1s., by post, 1s. 3d.**

To Become a **Good Nurse**

You must study the **Best Nursing Books.**

***Points Worth Remembering—***

**1. The Scientific Press, Ltd.,**

Publish Nursing Literature of all Descriptions.

NURSING TEXT-BOOKS,

TEMPERATURE CHARTS,

CASE-BOOKS, &c.

**2. The Scientific Press, Ltd.,**

Can supply all Works on

NURSING & KINDRED SUBJECTS

**(Both English and American),**

However difficult they may be to obtain elsewhere.

**3. The Scientific Press, Ltd.,**

Are Experts on

HOSPITAL & NURSING PUBLICATIONS,

And will be pleased to give Nurses information as to where they can obtain what they require.

**All Inquiries Receive Careful and Prompt Attention.**

*Note the Address of—*

THE NURSERY OF NURSING LITERATURE,

The Scientific Press, Ltd. **28 & 29 Southampton Street, Strand, London, W.C.**

**SEND A POSTCARD FOR NEW CATALOGUE OF NURSING MANUALS.**

